# Contributions of relative linear growth and adiposity accretion from birth to adulthood to adult hypertension

**DOI:** 10.1038/s41598-017-09027-1

**Published:** 2017-08-21

**Authors:** Alexandre Archanjo Ferraro, Marco Antônio Barbieri, Antonio Augusto Moura da Silva, Carlos Grandi, Viviane Cunha Cardoso, Aryeh D. Stein, Heloisa Bettiol

**Affiliations:** 1 Department of Pediatrics, Faculty of Medicine, University of Sao Paulo, Ribeirão Preto, Brazil; 2 Department of Pediatrics, Faculty of Medicine of Ribeirão Preto, University of São Paulo, Ribeirão Preto, Brazil; 3 Department of Public Health, University of Maranhão, São Luis, Brazil; 4 Department of Pediatrics, Faculty of Medicine, Universidad de Buenos Aires, Buenos Aires, Argentina; 5 Hubert Department of Global Health, Rollins School of Public Health, Emory University, Atlanta, GA USA

## Abstract

While birth weight and weight gain have been associated with hypertension (HT), the association of linear growth, independently of weight gains, has been less well studied. We assessed the independent association of body mass index (BMI) and length at birth and changes in BMI and height during the first two decades of life with adult blood pressure (BP). A birth cohort (n = 1141) was assembled in 1978–79, and followed up at school-age and adulthood. We used conditional length and BMI measures. BMI at birth was inversely associated with HT; c-BMI from school age to adulthood and c-height from birth to school age were positively associated with hypertension. Early adiposity accretion from birth to 9 years and late linear growth from 9 to 24 years were not associated with increased HT. Regarding BP, systolic and diastolic BP presented similar partterns: the lower the BMI at birth the higher the adult BP; the higher the BMI gains in the first 2 decades of life the higher the adult BP; linear accretion only in the first decade of life was associated with adult BP. Linear growth in the first decade of life and fat accretion in the second decade are associated with adults HT.

## Introduction

High blood pressure is widely prevalent in low and middle-income countries^1^. Many studies have shown an inverse association of birth weight with adult blood pressure^2, 3^. The Consortium of Health-Orientated Research in Transitioning Societies group found a small inverse association of birth weight, and a stronger positive association of postnatal weight gain, with systolic blood pressure, with no association of either birth weight or later weight gain with diastolic blood pressure^4^.

Birth weight is a limited indicator of fetal development^5^, and associations between length/height and body mass index with blood pressure have been less comprehensively examined. While height is correlated with blood pressure in cross-sectional models^6^, height gain has not been extensively studied; in one study height gain was positively associated with systolic but not with diastolic blood pressure at 10 years of age^7^, but studies have not extended into adulthood. Birth weight has most consistently been associated with systolic blood pressure in adulthood but associations with diastolic blood pressure are still inconclusive^2, 3^. Few studies have been conducted in low and middle-income settings where the burden of hypertension is emerging rapidly, and even fewer have taken a life-course perspective^4, 8^.

The relationship between linear growth and later blood pressure has been studied less frequently than the relationship between weight change in childhood and later blood pressure, and has not been studied previously using growth from birth to adulthood. Thus, the objective of the present study was to assess the independent associations of body mass index (BMI) and length at birth and changes in BMI and height from birth through childhood and from childhood through adulthood with adult HT and systolic and diastolic blood pressure, in a middle-income country setting.

## Methods

**Figure 1:**
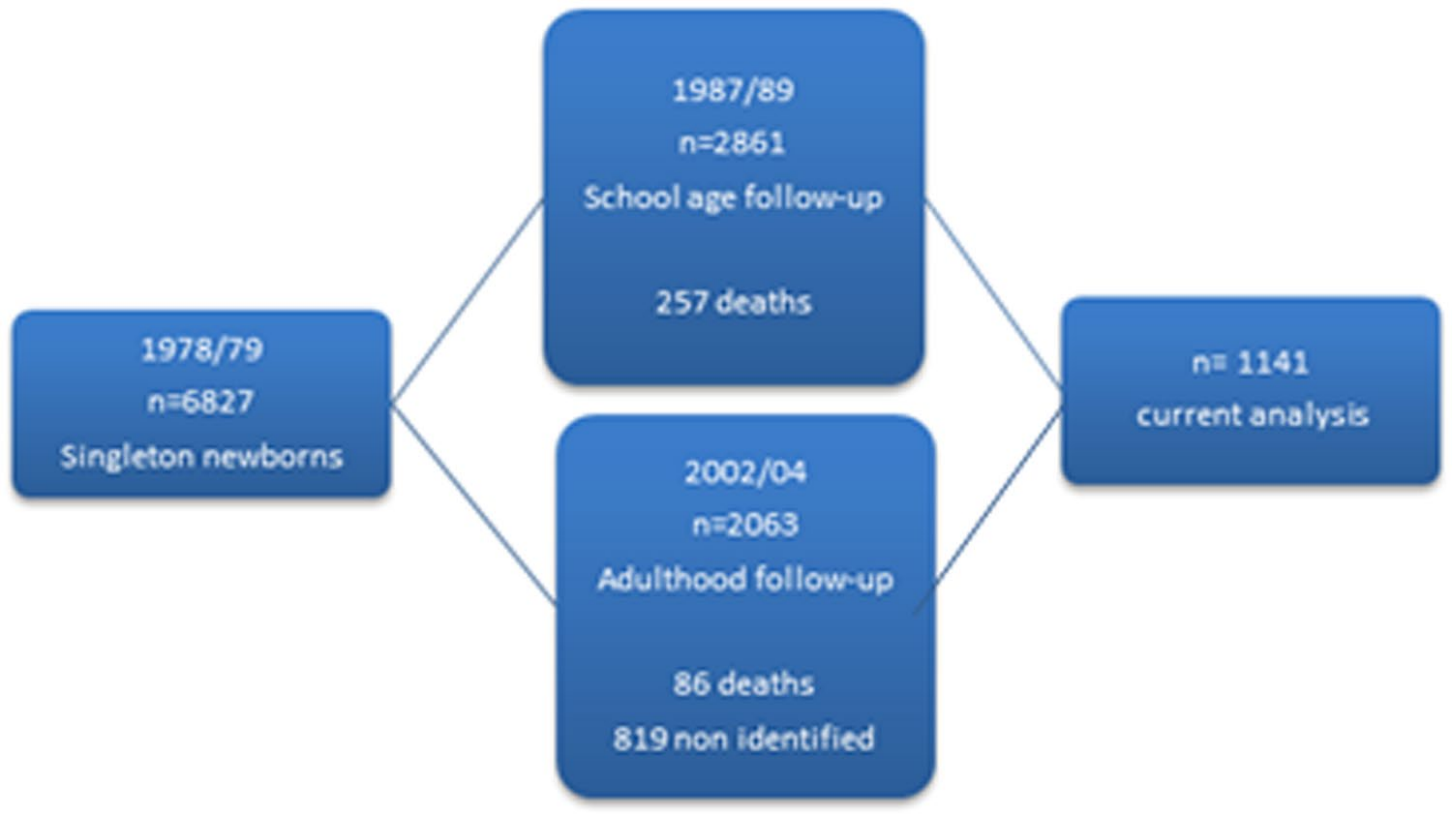
Flowchart of eligible population and sampling of the 1978/79 Ribeirao Preto birth cohort, Brazil.

Our data come from a prospective cohort study performed in the city of Ribeirão Preto, Brazil. The cohort was established in 1978/79, when 94.5% of all singleton live births to women resident in the city were recruited (n = 6827). In 1987–89, a school-age follow-up was conducted at child age 9–11 years. 2861 members of the cohort were identified, invited to participate, agreed and interviewed^9, 10^. In 2002/04, when individuals were aged 23–35 years, 5665 of the 6484 participants who were not known to have died were traced. A systematic 1 in 3 sample was performed (the first of every three names was selected from a list sorted by birth date in each geographic region and if unavailable, the next name down was selected) and 2063 cohort members were invited and reinterviewed (Fig. 1)^9, 11^. The sampling strategy at each follow up stage has been fully described elsewhere^10^.

Data from both the school and adult waves were available for 1141 individuals. This sample size has approximately 80% power to detect a risk ratio of 2.0, assuming the event has a 10% prevalence in the control group, with a 5% probability of type I error.

Maternal characteristics were collected by interview with the mother soon after birth. We extracted the following variables: ethnicity, occupation of the family head, based on the International Classification of Occupation^12^; maternal age at delivery in years (≤19, 20–34, ≥35); maternal schooling in years (≤4, 5–8, ≥9); number of cigarettes smoked per day during pregnancy (0, 1–10, >10); maternal hypertension during pregnancy; type of delivery (vaginal, cesarean section); gestational age in completed weeks based on the date of the last menstrual period. When unknown or implausible, missing gestational age was imputed in a regression model^13^, using birth weight, parity, family income and newborn gender. The newborn was considered preterm if the gestational age was <37 complete weeks. Maternal ethnicity was categorized as black and non-black based on self-reported skin colour.

Trained personnel measured newborn weight and length immediately after delivery. The babies were weighed naked in weekly-calibrated scales with 10 g precision. Two staff measured length with the baby laid in supine position on a neonatometer^14^.

At the school age examination, height and weight were measured according to standard techniques^15^.

As adults, the participants responded to a general questionnaire. Current occupation of the family head was classified in four categories: non-manual, skilled manual, semi-skilled manual, and unskilled manual. We administered the short version of the International Physical Activity Questionnaire (IPAQ)^16^ and individuals were classified as sedentary, insufficiently active and active. A food frequency questionnaire (FFQ) validated for the Brazilian population^17^ and applied by a nutritionist, ascertained information about intake over the last 12 months of 75 food items. The nutritional value of the diets was analyzed with the software Dietsys 4.0 (National Cancer Institute, Bethesda, MD, USA), and Brazilian foods and food preparations complementary to the Dietsys program were added when complete data about them were available from Brazilian tables^18^. Participants’ daily alcohol consumption was considered as none, low (≤31 g) and high (>31 g). The sodium intake considered only the existing amount of sodium in the consumed foods and was categorized as: <2000, 2000–2500, and >2500 mg/d. The number of cigarettes smoked per day was categorized as 0, 1–10, or >10.

Blood pressure was measured in triplicate^19^ using a 740 Omron digital sphygmomanometer (OMRON Healthcare Europe B.V., Hoofdorp, The Netherlands) with a single-size cuff that was adjusted to the arm circumference. One person made all measurements at 15-min intervals, with the participant resting in sitting position with the left arm at the height of the heart. The mean of the last two measurements was use in analysis. Individuals with a systolic blood pressure above 140 and/or a diastolic blood pressure above 90 were considered as hypertensive as were those individuals who were taking medications for hypertension.

Weight was measured with an adult electronic scale (Filizola®, Sao Paulo, Brazil) with 100 g precision. Height was measured to the nearest centimeter using a stadiometer, according to standard techniques^15^.

Ethical approval was given by all maternities and by the Research Ethics Committee of the University Hospital, Faculty of Medicine of Ribeirão Preto, University of São Paulo (protocol no. 7606/99). All participants provided their written informed consent to participate in this study. These consents were approved by the ethics committee and one copy was given to the participant and one copy was kept with the research team.

Mean and standard deviation were used to describe the Body Mass Index (BMI) and height according to sex and age. Systolic and diastolic blood pressure were described according to sex. Groups were compared using the t test. All variables were tested for normality.

BMI and height were standardized for age and sex using ANTHRO® and ANTHROPLUS®, which use the World Health Organization reference data. Since ANTHROPLUS® only goes to age 19 years, we considered the value at this age as the final adult height.

In order to eliminate the problem of collinearity among the three repeated measures of BMI or height, we computed conditional growth measures^4, 20^. These are calculated as the residuals from linear regressions of BMI and height at a given age and gender on all earlier measures of BMI and height. A positive residual indicates that a child grew more rapidly during the interval than would have been predicted based on its initial values and prior growth.

Frequencies were used to describe data according to the presence of hypertension or not in adulthood. Groups were compared through chi-square tests. We used Poisson regression to estimate incidence rate ratio (IRR) for HT and linear regression for systolic and diastolic BP^21^. To deal with missing data we chose to perform inverse probability weighting, weighting for all variables that were linked to loss of follow up from birth to adulthood^22^.

To avoid overadjustment covariates were selected with the aid of a directed acyclic graph (DAG)^23^ (Supporting Information Figure S1), following current practice in life course epidemiology, which highlights the risks of overadjustment by factors that might be intermediate in the pathways under consideration, and are therefore not confounders. Each variable was ordered according to its specific temporal relationship: pre-conception (maternal skin colour; occupation of the head of the family, maternal age and schooling); antenatal (child’s gender and maternal smoking during pregnancy); perinatal (maternal hypertension at delivery, type of delivery; BMI, length and gestational age); school age (type of school; BMI and height); adulthood (adult age, occupation of the head of the family; physical activity, smoking, alcohol consumption, sodium intake; blood pressure, BMI and height). Identifying temporal order facilitates understanding relationship between variables. We then built pathways based on temporality and theoretical assumptions. The resulting model was built upon these associations in a browser-based environment (http://www.dagitty.net). Adult risk factors for HT (physical activity, alcohol and sodium intake) did not fulfill the criteria do be considered confounders, because they are not antecedents of the exposures. This approach to deal with confounding covariates is increasingly being used^24–26^.

Since the final number of covariables was large, we double checked the results by bootstrapping the adjusted models to ensure results were robust (Supporting Information Tables S1 and S2).

Since gender differences in effects of adiposity on blood pressure have been reported^27^, interaction terms were tested between gender and all six growth parameters in the Poisson regression models. Finally, to check for a U-shape association between anthropometric variables and HT/blood pressure we included quadratic terms for each of the six growth parameters in the regression models (Supporting Information Table S3). Those that were significant were included in the tables. The STATA 10.1® statistical package was used for all analyses and significance level was set at 0.05.

## Results

The studied sample differs in some aspects from the original birth cohort. Participants with low birth weight or low length at birth, born vaginally, from mothers with low schooling, smokers or low maternal age or whose heads of family were engaged in manual occupations were less likely to be followed-up (Table 1). Men had higher length at birth, higher blood pressure, higher BMI and height in adulthood and were more frequently hypertensive than women (Table 2). Preterm babies (6.1%) had a mean gestational age of 35.2 (sd 1.2). Eighteen mothers (1.6%) presented HT at delivery. Type of delivery (c-section), gender (males), smoking, greater alcohol and sodium intakes were associated with HT (Table 3).

**Table 1:** Comparison of the characteristics at birth of non followed up subjects with those followed up until adulthood (2002/2004).

Variables	Categories	Not followed up N = 5343*	Followed up N = 1141	p-value
Total		%	%	
Occupation of the				<0.001
head of the family	unskilled	24.8	16.5	
	skilled and semi-skilled	55.3	64.2	
	non manual	16.6	16.7	
	unknown	3.3	2.6	
Maternal age (years)				0.005
	<20	14.5	11.0	
	20–34	77.0	81.2	
	> = 35	8.5	7.8	
Maternal schooling (years)				<0.001
	0 to 4	50.8	41.2	
	5 to 11	36.8	45.1	
	> = 12	9.7	12.0	
	unknown	2.6	1.7	
Maternal smoking				<0.001
	yes	29.9	23.0	
	no	70.1	77.0	
Gender				0.072
	male	51.3	48.6	
	female	48.5	51.4	
Prematurity				0.815
	yes	5.9	6.1	
	no	94.1	93.9	
Type of birth				0.019
	vaginal	70.3	66.8	
	c-section	29.7	33.2	
Birth weight (g)				0.007
	<2500	6.2	4.2	
	2500–2999	21.3	18.3	
	3000–3499	40.3	43.0	
	3500–3999	25.6	26.9	
	> = 4000	6.6	7.5	
Birth length (cm)				0.003
	<47	11.4	9.8	
	47–48.9	25.0	24.2	
	49–50.1	41.3	40.0	
	51–52.9	17.4	20.9	
	> = 53	4.2	5.1	
	unknown	0.6	0.0	

*343 deaths excluded.

**Table 2:** Body mass index (BMI), length/height and blood pressure, in a population-based birth cohort born in 1978–79 in Ribeirao Preto, Brazil, by sex.

	Unit	MALES N = 551	FEMALES N = 590
		mean (sd)	mean (sd)
BMI at birth	kg/m^2^	13.6 (1.5)	13.5 (1.4)
	z-score	0.1 (1.1)	0.1 (1.1)
BMI at 8–11 y	kg/m^2^	17.2 (3.0)	17.1 (2.8)
	z-score	0.2 (1.3)	0.1 (1.2)
BMI at 23–25 y	kg/m^2^	25.2 (5.5)*	23.6 (5.7)
	z-score	0.7 (1.2)*	0.4 (1.2)
Length at birth	cm	49.7 (2.2)*	48.9 (2.1)
	z-score	−0.1 (1.1)	−0.1 (1.2)
Height at school age	cm	136.8 (6.5)	137.1 (7.0)
	z-score	0.1 (1.0)	−0.0 (1.1)
Height in adulthood	cm	176.3 (6.5)*	162.9 (6.4)
	z-score	−0.0 (0.9)	−0.1 (1.0)
Systolic blood pressure	mm Hg	126.5 (13.0)*	108.9 (11.2)
Diastolic blood pressure	mm Hg	74.1 (8.8)*	67.7 (8.2)
Prevalence of hypetension	%	16.5 (13.4–19.6)**	3.1 (1.7–4.4)**

sd = standard deviation. *t test p < 0.001; **chi-square test < 0.001; 95% confidence interval for the prevalence estimates.

**Table 3:** Selected characteristics of a population–based birth cohort born in 1978–79 in Ribeirao Preto, Brazil, by hypertension status in adulthood.

BIRTH	NON HT (n = 1032)	HT (n = 109)	p
	%	%	
***Maternal Hypertension***			0.83
no	98.4	98.2	
***Type of Delivery***			0.02
vaginal	67.7	56.9	
***Maternal Smoking***			0.5
no	81.0	84.0	
1–10 /day	14.3	12.2	
+ 10/day	4.7	3.8	
***Maternal age (years)***			0.1
< = 19	11.2	8.3	
20–34	81.4	80.7	
> = 35	7.4	11.0	
***Maternal Schooling (years)***			0.3
0–4	42.5	35.8	
5–8	27.7	34.0	
9 or +	29.8	30.2	
***Occupation of the family head***			0.5
Non qualified manual	16.9	16.9	
Semi-qualified manual	66.1	64.2	
Non manual + qualified manual	17.0	18.9	
***Maternal skin colour***			0.3
Non black	70.4	65.2	
***Preterm birth***			0.8
yes	6.2	5.5	
***Gender***			<0.001
male	44.6	83.5	
female	55.4	16.5	
**School age**			
***Type of school***			0.8
Private	15.8	14.3	
Fee-free private	13.5	11.6	
Public	70.7	74.1	
**Adulthood**			
***Physical activity***			0.067
sedentary	50.1	42.2	
sufficient	19.0	21.1	
high	30.9	36.7	
***Smoking***			0.048
no	84.4	80.7	
1–10 /day	10.3	6.4	
+10/day	5.3	12.9	
***Alcohol intake***			<0.001
none	25.9	16.5	
low	56.0	45.0	
high	18.1	38.5	
***Sodium intake (mg/day)***			0.005
≤2000	36.2	23.9	
2000–2500	26.8	26.6	
>2500	37.0	49.5	
Occupation of the family head			0.715
Non qualified manual	15.3	14.8	
Semi-qualified manual	20.8	21.3	
Qualified manual	27.4	25.0	
Non manual	36.5	38.9	

HT = hypertension; statistic test: chi-square or chi-square for trend tests.

Table 4 shows that neither birth length nor BMI at birth were associated with HT; at school age height and conditional height were positively associated with HT, with no association observed for BMI; adult BMI and height were associated with HT whether expressed in absolute or conditional units.

**Table 4:** Anthropometric variables at birth, school age and adulthood in a population–based birth cohort born in 1978–79 in Ribeirao Preto, Brazil, by hypertension status in adulthood.

BIRTH	NON HT (n = 1032)	HT (n = 109)	p
	mean (sd)	mean (sd)	
BMI (kg/m^2^)	13.57 (1.43)	13.38 (1.38)	0.179
BMI (z-score)	0.09 (1.13)	−0.09 (1.08)	0.120
Length (cm)	49.25 (2.16)	49.53 (2.21)	0.195
Length (z-score)	−0.12 (1.12)	−0.12 (1.18)	0.973
**School age**			
BMI (kg/m^2^)	17.08 (2.85)	17.62 (3.36)	0.064
c-BMI (z-score)	−0.01 (0.99)	0.12 (1.09)	0.205
Height (cm)	136.71 (6.72)	138.21 (6.79)	0.027
c-Height (z-score)	−0.02 (1.00)	0.20 (0.98)	0.032
**Adulthood**			
BMI (kg/m^2^)	23.89 (4.30)	27.98 (6.30)	<0.001
c-BMI (z-score)	−0.08 (0.96)	0.75 (1.09)	<0.001
Height (cm)	168.71 (9.13)	175.59 (8.81)	<0.001
c-Height (z-score)	−0.02 (0.99)	0.20 (1.10)	0.026

HT = hypertension; BMI = body mass index; c-BMI = conditional BMI; c-height = conditional height; statistic test: t-test.

The associations of growth (BMI and height) in specific life stages with HT are shown in Table 5. The quadratic conditional-height from school-age to adulthood was significant only when it was not adjusted for the other anthropometric variables (model 1). In the final adjusted model 2, BMI at birth was inversely associated with HT (IRR = 0.79 [0.66–0.95]) and conditional BMI from school age to adulthood was positively associated with HT (IRR = 1.83 [1.55–2.16]). Conditional height from birth to school age was positively associated with HT (IRR = 1.40 [1.16–1.69]). Early adiposity accretion from birth to 9 years and late linear growth from 9 to 24 years were not associated with increased HT. The results are presented separately for men and women in the Supporting Information Table S4 (there was no statistically significant heterogeneity by sex).

**Table 5:** Poisson regression of the association of growth patterns and hypertension in a population–based birth cohort born in 1978–79 in Ribeirao Preto, Brazil (N = 1141).

unit: z-score	non adjusted			adjusted model 1			adjusted model 2		
	RR	95%CI	p	RR	95%CI	p	RR	95%CI	p
BMI at birth	0.90	0.75–1.08	0.255	0.82	0.68–0.98	0.034	0.79	0.66–0.95	0.013
conditional BMI at school	1.13	0.92–1.38	0.237	1.04	0.87–1.24	0.686	1.15	0.97–1.36	0.117
conditional BMI in adulthood	1.92	1.65–2.23	<0.001	1.85	1.57–2.17	<0.001	1.83	1.55–2.16	<0.001
length at birth	1.16	0.95–1.41	0.147	0.99	0.81–1.21	0.957	1.03	0.85–1.25	0.748
conditional height at school	1.26	1.06–1.48	0.007	1.33	1.12–1.57	0.001	1.40	1.16–1.69	<0.001
conditional height in adulthood	1.16	0.96–1.42	0.131	1.14	0.96–1.36	0.147	1.12	0.93–1.36	0.230
quadratic adult cond. height	—	—	—	1.07	1.02–1.12	0.005	—	—	—

Unadjusted model: each anthropometric measure entered into a separate model. Model 1 is adjusted for for gender, type of delivery, preterm birth, maternal hypertension, adult smoking, adult age and adult occupation of the head of the family. Model 2 includes all anthropometric variables entered together in model I. *IRR* = incidence rate ratio; *c-BMI* = conditional BMI; *c-length* = conditional height; *95%CI* = 95% confidence interval.

Table 6 presents the results for systolic and diastolic BP. All variables except length at birth and conditional height between 9 and 24 years were positively associated with systolic and diastolic blood pressure (BMI at birth was inversely associated).

**Table 6:** Linear regression of the association between growth patterns and systolic and diastolic blood pressure in a population–based birth cohort born in Brazil (N = 1141).

unit: z-score	non adjusted			adjusted model 1			adjusted model 2		
	*beta*	*95%CI*	*p*	*beta*	*95%CI*	*p*	*beta*	*95%CI*	*p*
**Systolic blood pressure**									
BMI at birth	−0.31	−1.22/0.61	0.510	−0.80	−1.55/−0.04	0.040	−0.87	−1.60/−0.14	0.019
conditional BMI at school	1.18	0.22/2.14	0.016	0.72	−0.03/1.46	0.060	1.29	0.56/2.02	0.001
conditional BMI in adulthood	4.41	3.54/5.28	<0.001	3.32	2.60/4.04	<0.001	3.26	2.54/3.98	<0.001
length at birth	1.71	0.86/2.56	<0.001	0.07	−0.64/0.78	0.844	0.17	−0.51/0.86	0.621
conditional height at school	1.17	0.31/2.04	0.008	1.41	0.69/2.12	<0.001	1.76	1.04/2.47	<0.001
conditional height in adulthood	0.53	−0.41/1.47	0.266	0.73	−0.01/1.48	0.055	0.42	−0.28/1.109	0.238
**Diastolic blood pressure**									
BMI at birth	−0.28	−0.85/0.30	0.345	−0.43	−0.97/0.10	0.110	−0.49	−0.97/−0.01	0.045
conditional BMI at school	1.44	0.91/1.98	<0.001	1.31	0.82/1.80	<0.001	1.80	1.33/2.27	<0.001
conditional BMI in adulthood	3.64	3.11/4.17	<0.001	3.28	2.77/3.786	<0.001	3.29	2.80/3.79	<0.001
length at birth	0.77	0.21/1.33	0.007	0.22	−0.31/0.74	0.420	0.31	−0.17/0.78	0.204
conditional height at school	0.97	0.44/1.50	<0.001	1.00	0.50/1.50	<0.001	1.49	1.01/1.97	<0.001
conditional height in adulthood	0.14	−0.48/0.75	0.664	0.16	−0.42/0.73	0.594	−0.18	−0.67/0.32	0.487

Unadjusted model: each anthropometric measure entered into a separate model. Model 1 BMI and length/height entered into separate models that were adjusted for gender, type of delivery, gestational age, maternal hypertension, adult smoking, adult age and occupation of the head of the family in adulthood. Model 2 BMI and length/height were adjusted for each other and for gender, type of delivery, gestational age, maternal hypertension, adult smoking, adult age and occupation of the head of the family in adulthood. *95%CI* = 95% confidence interval.

## Discussion

We found that lower BMI at birth was associated with higher adult HT. Higher than expected height gain in the first decade of life was associated with adult HT but higher than expected gain in the second decade of life was not. On the other hand, higher than expected BMI gain in the second decade of life was associated with HT but higher than expected BMI gain during the first decade of life was not. Regarding BP, systolic and diastolic BP presented similar partterns: BMI at birth was associated inversely and BMI gains in the first 2 decades of life positively; linear accretion only in the first decade of life was associated with BP.

Our findings suggest that linear growth in early childhood, and increases in adiposity later in children, are important for risk of HT. The relationship between linear growth and later blood pressure has been studied less frequently than has the relationship between weight change in childhood and later blood pressure.

Faster growth has been related to a range of positive and adverse outcomes. Huang *et al*.^28^ showed the importance of postnatal weight gain on blood pressure in mid childhood. Adair *et al*.^4^ demonstrated the importance of separating the effect of weight growth and linear growth on adult outcomes: on one hand, rapid height gain in the first years of life was associated with increased schooling and final height. Conversely, greater than expected weight gain in later childhood was linked to obesity, increases in systolic blood pressure and glucose impairment. However, Adair *et al*. do not have an age period that dichotomizes at the immediate pre-pubertal period.

In the context of blood pressure, taller adults have higher BP, but reduced risk for CVD^29^. Should we favor a rapid growth of a small for gestational age babies? Which growth parameter is best?

Our study helps to clarify some of these issues. Inverse associations of BMI at birth with hypertension are evident in young adulthood. This is in line with amounting evidence^2, 3^. As was also seen in the ALSPAC study^30^ length at birth was not related to adult blood pressure. However, we found stronger associations with hypertension for height gain in the first decade of life and weight gain in the second decade of life than in this study. These associations are stronger and positive compared with the inverse association of BMI at birth with hypertension. This could suggest that postnatal growth is more strongly associated with hypertension than is prenatal growth^7^.

It has been pointed out that increases in relative body size are more predictive of adult blood pressure after age 11 years than in earlier childhood^31^. Moreover, there is evidence that growth in early life (first 48 months) has less influence in BP than growth from 48 months to adulthood^32^. Our results are also consistent with these strengthening associations of weight gain from birth to young adulthood with adult hypertension^30, 31^. As others had already seen^33^, higher than expected BMI gain seems to be deleterious to adult blood pressure and this association seems to amplify across infancy and adolescence until early adulthood. We found that higher than expected BMI gain in the second decade of life was associated with higher blood pressure whereas BMI gain in the first decade of life was not. In addition, with simultaneous adjustment for weight and length/height gain the associations of weight gain on systolic and diastolic blood pressure were of similar magnitude. In ALSPAC, however, associations of BMI gain on DBP were weaker than those with SBP^7^.

Since height is related to BP, why then is height change in the period from childhood to adulthood not associated with adult BP? As has been demonstrated elsewhere^34^ differences in adult height are already evident at the earlier time points. Our results are consistent with decreasing or even disappearing associations of height gain on adult hypertension because height gain was only moderately associated with SBP and not with DBP or hypertension in the second decade of life. In addition, association between height gain and SBP reduced from the first to the second decade of life. Thus, hypertension due to accelerated gains in height in early life seems not to persist through adult life. This decreasing or even vanishing association of height gain on adult hypertension seems to represent a physiological rather than a pathological effect. It might be explained by a mismatch between linear and renal/arteriolar growth and development^35^. It seems that in infancy and post-infancy, early linear growth spurts are not accompanied by similar developments in renal and arteriolar function, thus resulting in higher blood pressure. Our results suggest that this adaptative mechanism that results in higher blood pressure in early life due to the linear growth spurt are transient and do not track to adult life. In contrast, effects of BMI gain seem to persist and increase from birth to adulthood.

In agreement with our data, results from a pooled analysis of birth cohort studies from low and middle income countries also reported that height gains at age 2 years and mid-childhood but not in adulthood were associated with SBP^4^. However, Menezes reported that early length/height gains in the first four years of life are not associated with higher systolic blood pressure^36^.

It is important to note that in our study the association between height gain and diastolic blood pressure was only evident from birth to school age and was weaker than the corresponding association with systolic blood pressure. This is in contrast to ALSPAC data, in which diastolic BP at 10 years was not influenced by height growth^7^.

The present study has some strengths worth underlying: it is a population-based prospective birth cohort from a middle-income setting. The conditional modeling of height and BMI changes provides information on the relative contribution of BMI gain and linear growth in predicting adult blood pressure.

A limitation of this study is measurement error. Newborn length is not always precisely measured. We minimized the potential for error by standardizing data collection. Blood pressure assessed at one point in time may be elevated because of white coat syndrome. This would affect the whole sample, resulting in non-differential misclassification. Another limitation is that losses to follow-up may have generated a sample with better health and social conditions than the original subjects at birth. If we had had access to these less healthy and wealthy subjects our effect size would possibly be stronger. However, we performed inverse probability weighting to minimize this bias. An important limitation is that no data are available on growth from birth to 2 years^34^.

The independent association of growth patterns with adult hypertension, even after controlling for adult risk factors confirms that CVD prevention actions should not be restricted to adulthood. This evidence has been acknowledged by the United Nations heads of States and governments^37^.

In conclusion, postnatal growth seems to have more influence on hypertension than prenatal growth. Higher BP due to accelerated gains in height in early life seems to be an evidence for the first decade of life, but it does not track into the second one. Higher than expected fat accretion seems to be deleterious to adult blood pressure and its effect seems to be bigger in the second than in the first decade of life. Strategies for preventing fat accretion in the first and especially in the second decade of life are probably more important than strategies focusing on early mother and child nutrition to prevent hypertension in adulthood.

## Electronic supplementary material

Supporting Information
