# Supplementary material for: Contributions of relative linear growth and adiposity accretion from birth to adulthood to adult hypertension
Source: Sci Rep. 2017 Aug 21;7:8928. doi: 10.1038/s41598-017-09027-1 (PMC5566373; doi:10.1038/s41598-017-09027-1)
Supplement: Supplementary file 1 — Supporting Information [file 41598_2017_9027_MOESM1_ESM.pdf]

SUPPORTING INFORMATION

Contributions of relative linear growth and adiposity accretion from birth to adulthood to adult hypertension

Alexandre Archanjo Ferraro (1), Marco Antônio Barbieri (2), Antonio Augusto Moura da Silva (3), Carlos Grandi (4), Viviane Cunha Cardoso (2), Aryeh D Stein (5), Heloisa Bettiol (2)

AUTHORS' AFFILIATIONS:

(1) - Department of Pediatrics - Faculty of Medicine - University of Sao Paulo, Ribeirão Preto, Brazil;

(2) - Department of Pediatrics, Faculty of Medicine of Ribeirao Preto, University of Sao Paulo, Ribeirão Preto, Brazil;

(3) - Department of Public Health, University of Maranhão, São Luis, Brazil

(4) - Department of Pediatrics, Faculty of Medicine, Universidad de Buenos Aires, Buenos Aires, Argentina

(5) - Hubert Department of Global Health, Rollins School of Public Health, Emory University, Atlanta, GA

S1 Figure: DIRECTED ACYCLIC GRAPHS

Minimal Sufficient Adjustment Set for estimating the total effect of anthropometric exposures on blood pressure: gestational age, maternal hypertension, type of delivery, gender, adult age, adult smoking and occupation of the head of the family in adulthood.

Legend: AdultAge; AdultOccup (occupation of the head of the family in adulthood); AgeSchool (age at school assessment); Alcohol (adult alcohol intake); BMIBirth (body mass index at birth); C-BMIAdult (conditional body mass index in adulthood); C-BMISchool (conditional body mass index in school age); C-HeightAdult (conditional height in adulthood); C-HeightSchool (conditional height in school age); GestAge (gestational age at birth); HighBloodPres (high blood pressure); LengthBirth (length at birth); MatAge (maternal age at delivery time); MatHypertens (maternal hypertension at delivery); MatSchool (maternal schooling); MatSkin (maternal skin colour); MatSmoke (maternal smoking); NaIntake (adult sodium intake); OccupAtBirth (occupation of the head of the family at birth); PhysAct (adult physical activity); Smoking (adult smoking); TypeDelivery; TypeSchool; gender

S1 Table: bootstrap replications (100) - Linear regression of the association between growth parameters and systolic blood pressure

Model 1 BMI and length/height entered into separate models that were adjusted for for gender, type of delivery, gestational age, maternal hypertension, adult smoking, adult age and occupation of the head of the family in adulthood. Model 2 BMI and length/height were adjusted for each other and for gender, type of delivery, gestational age, maternal hypertension, adult smoking, adult age and occupation of the head of the family in adulthood; *95%CI* =95%

S2 Table: bootstrap replications (100) - Linear regression of the association between growth parameters and diatolic blood pressure

Model 1 BMI and length/height entered into separate models that were adjusted for for gender, type of delivery, gestational age, maternal hypertension, adult smoking, adult age and occupation of the head of the family in adulthood. Model 2 BMI and length/height were adjusted for each other and for gender, type of delivery, gestational age, maternal hypertension, adult smoking, adult age and occupation of the head of the family in adulthood; *95%CI* =95%

S3 Table: Test for quadratic distribution

* Poisson multivariate models adjusted for gender, gestational age, maternal hypertension, type of delivery, maternal smoking, age at adult assessment, occupation of the head of the family in adulthood.
** same as previous model, but with growth parameters adjusted for one another

S4 Table: Poisson regression of the association of growth patterns and hypertension according to sex, in a population–based birth cohort born in 1978-79 in Ribeirao Preto, Brazil (N=1141).

Unadjusted model: each anthropometric measure entered into a separate model. Model is adjusted for for type of delivery, preterm birth, maternal hypertension, adult smoking, adult age and adult occupation of the head of the family and all anthropometric variables entered together. *IRR*= incidence rate ratio; *c-BMI*=conditional BMI; *c-length*=conditional height; *95%CI* =95% confidence interval.

Supplemental Figure S2: DIRECTED ACYCLIC GRAPHS

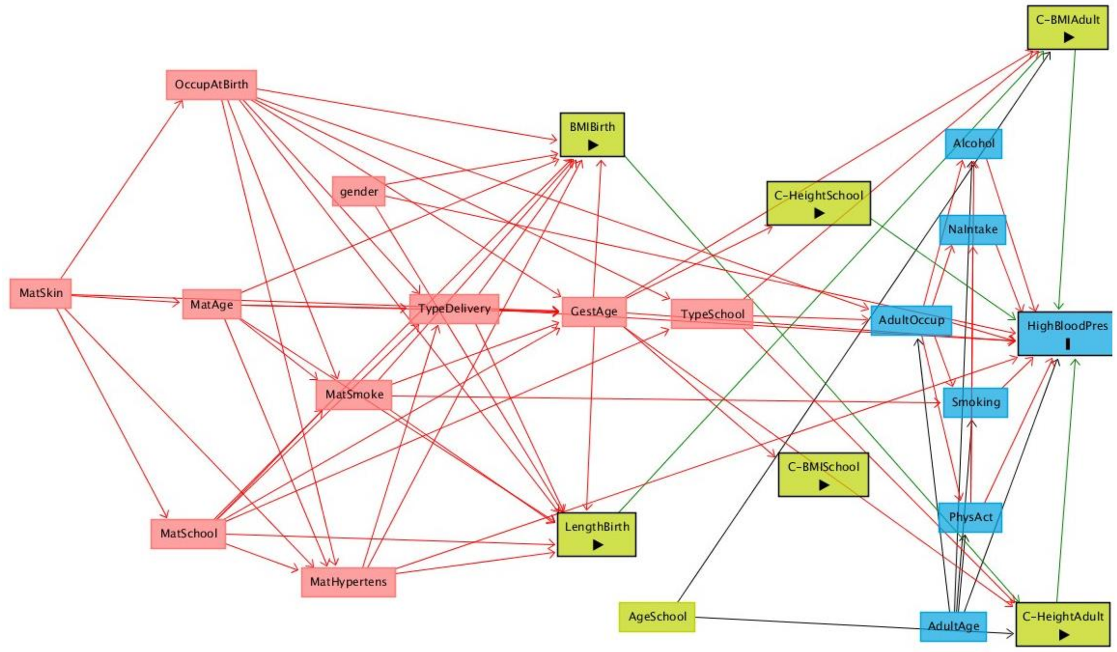

exposure

outcome

ancestor of exposure

ancestor of outcome

ancestor of exposure *and* outcome

causal path

biasing path

<u>Minimal Sufficient Adjustment Set</u> for estimating the total effect of anthropometric exposures on blood pressure: gestational age, maternal hypertension, type of delivery, gender, adult age, adult smoking and occupation of the head of the family in adulthood.
<u>Legend</u>: AdultAge; AdultOccup (occupation of the head of the family in adulthood); AgeSchool (age at school assessment); Alcohol (adult alcohol intake); BMIBirth (body mass index at birth); C-BMIAdult (conditional body mass index in adulthood); C-BMISchool (conditional body mass index in school age); C-HeightAdult (conditional height in adulthood); C-HeightSchool (conditional height in school age); GestAge (gestational age at birth); HighBloodPres (high blood pressure); LengthBirth (length at birth); MatAge (maternal age at delivery time); MatHypertens (maternal hypertension at delivery); MatSchool (maternal schooling); MatSkin (maternal skin colour); MatSmoke (maternal smoking); NaIntake (adult sodium intake); OccupAtBirth (occupation of the head of the family at birth); PhysAct (adult physical activity); Smoking (adult smoking); TypeDelivery; TypeSchool; gender

Supporting Information S1 Table: bootstrap replications (100) - Linear regression of the association between growth parameters and systolic blood pressure

	Model 1		Model 2	
**Growth parameter**	**Observed coeffitient**	**Normal-based 95% CI**	**Observed coeffitient**	**Normal-based 95% CI**
BMI at birth	-1.08	-1.83 / -0.32	-1.13	-1.76 / -0.51
BMI at schoolage	0.81	0.13 / 1.48	1.37	0.66 / 2.08
BMI in adulthood	3.43	2.82 / 4.04	3.38	2.78 / 3.98
Length at birth	-0.15	-0.82 / 0.53	-0.09	-0.76 / 0.58
Height at schoolage	1.34	0.56 / 2.12	1.75	1.15 / 2.36
Height in adulthood	0.75	0.05 / 1.45	0.44	-0.19 / 1.08

Model 1 BMI and length/height entered into separate models that were adjusted for for gender, type of delivery, gestational age, maternal hypertension, adult smoking, adult age and occupation of the head of the family in adulthood. Model 2 BMI and length/height were adjusted for each other and for gender, type of delivery, gestational age, maternal hypertension, adult smoking, adult age and occupation of the head of the family in adulthood; *95%CI* =95%

Supporting Information S2 Table: bootstrap replications (100) - Linear regression of the association between growth parameters and diatolic blood pressure

	Model 1		Model 2	
**Growth parameter**	**Observed coeffitient**	**Normal-based 95% CI**	**Observed coeffitient**	**Normal-based 95% CI**
BMI at birth	-0.51	-1.03 / 0.00	-0.55	-0.97 / -0.13
BMI at schoolage	1.30	0.88 / 1.74	1.80	1.38 / 2.21
BMI in adulthood	3.27	2.77 / 3.77	3.29	2.82 / 3.76
Length at birth	0.19	-0.33 / 0.71	0.23	-0.24 / 0.71
Height at schoolage	0.96	0.45 / 1.47	1.49	1.09 / 1.88
Height in adulthood	0.20	-0.39 / 0.78	-0.13	-0.63 / 0.37

Model 1 BMI and length/height entered into separate models that were adjusted for for gender, type of delivery, gestational age, maternal hypertension, adult smoking, adult age and occupation of the head of the family in adulthood. Model 2 BMI and length/height were adjusted for each other and for gender, type of delivery, gestational age, maternal hypertension, adult smoking, adult age and occupation of the head of the family in adulthood; *95%CI* =95%

Supporting Information S3 Table: Test for quadratic distribution

	p (Wald test)		
**Growth parameter**	**Bivariate model**	**Multivariate model ***	**Multivariate model ****
BMI at birth	0.380	0.324	-
BMI at schoolage	0.100	0.421	-
BMI in adulthood	0.392	0.836	-
Length at birth	0.667	0.846	-
Height at schoolage	0.236	0.461	-
Height in adulthood	0.122	0.005	0.187

* Poisson multivariate models adjusted for gender, gestational age, maternal hypertension, type of delivery, maternal smoking, age at adult assessment, occupation of the head of the family in adulthood.
** same as previous model, but with growth parameters adjusted for one another

Supporting Information S4 Table: Poisson regression of the association of growth patterns and hypertension according to sex, in a population–based birth cohort born in 1978-79 in Ribeirao Preto, Brazil (N=1141).

	**adjusted model**		
	IRR	95%CI	p
**MEN**			
**BMI at birth**	0.84	0.70-1.00	0.054
**conditional BMI at school**	1.07	0.89-1.31	0.466
**conditional BMI in adulthood**	1.71	1.42-2.05	<0.001
**length at birth**	0.98	0.81-1.18	0.815
**conditional height at school**	1.33	1.08-1.64	0.007
**conditional height in adulthood**	1.22	1.00-1.48	0.051
**WOMEN**			
**BMI at birth**	0.58	0.35-0.98	0.041
**conditional BMI at school**	1.37	0.87-2.14	0.173
**conditional BMI in adulthood**	2.59	1.75-3.82	<0.001
**length at birth**	1.22	0.78-1.90	0.377
**conditional height at school**	1.83	1.20-2.79	0.005
**conditional height in adulthood**	0.80	0.57-1.12	0.200

Unadjusted model: each anthropometric measure entered into a separate model. Model is adjusted for for type of delivery, preterm birth, maternal hypertension, adult smoking, adult age and adult occupation of the head of the family and all anthropometric variables entered together. *IRR*= incidence rate ratio; *c-BMI*=conditional BMI; *c-length*=conditional height; *95%CI* =95% confidence interval.
